# Transcriptomic and Metabolomic Analysis Revealed Multifaceted Effects of Phage Protein Gp70.1 on *Pseudomonas aeruginosa*

**DOI:** 10.3389/fmicb.2016.01519

**Published:** 2016-09-26

**Authors:** Xia Zhao, Canhuang Chen, Xingyu Jiang, Wei Shen, Guangtao Huang, Shuai Le, Shuguang Lu, Lingyun Zou, Qingshan Ni, Ming Li, Yan Zhao, Jing Wang, Xiancai Rao, Fuquan Hu, Yinling Tan

**Affiliations:** ^1^Department of Microbiology, Third Military Medical UniversityChongqing, China; ^2^No.180 Hospital of PLAQuanzhou City, China; ^3^Department of Clinical Laboratory, Xinqiao Hospital, Third Military Medical UniversityChongqing, China; ^4^Institute of Burn Research, Southwest Hospital, Third Military Medical UniversityChongqing, China

**Keywords:** *Pseudomonas aeruginosa*, bacteriophage, phage–host interaction, host shut-off proteins, RpoS

## Abstract

The impact of phage infection on the host cell is severe. In order to take over the cellular machinery, some phage proteins were produced to shut off the host biosynthesis early in the phage infection. The discovery and identification of these phage-derived inhibitors have a significant prospect of application in antibacterial treatment. This work presented a phage protein, gp70.1, with non-specific inhibitory effects on *Pseudomonas aeruginosa* and *Escherichia coli.* Gp70.1 was encoded by early gene – *orf 70.1* from *P. aeruginosa* phage PaP3. The *P. aeruginosa* with a plasmid encoding gp70.1 showed with delayed growth and had the appearance of a small colony. The combination of multifaceted analysis including microarray-based transcriptomic analysis, RT-qPCR, nuclear magnetic resonance (NMR) spectroscopy-based metabolomics and phenotype experiments were performed to investigate the effects of gp70.1 on *P. aeruginosa*. A total of 178 genes of *P. aeruginosa* mainly involved in extracellular function and metabolism were differentially expressed in the presence of gp70.1 at three examined time points. Furthermore, our results indicated that gp70.1 had an extensive impact on the extracellular phenotype of *P. aeruginosa*, such as motility, pyocyanin, extracellular protease, polysaccharide, and cellulase. For the metabolism of *P. aeruginosa*, the main effect of gp70.1 was the reduction of amino acid consumption. Finally, the RNA polymerase sigma factor RpoS was identified as a potential cellular target of gp70.1. Gp70.1 was the first bacterial inhibitor identified from *Pseudomonas aeruginosa* phage PaP3. It was also the first phage protein that interacted with the global regulator RpoS of bacteria. Our results indicated the potential value of gp70.1 in antibacterial applications. This study preliminarily revealed the biological function of gp70.1 and provided a reference for the study of other phage genes sharing similarities with *orf70.1*.

## Introduction

Bacteriophage (phage) is an obligate intracellular parasite and it exploits the host cell’s resources including intracellular environment, enzymes, and energy, for propagation. During the long co-evolutionary history with their host, phage develops unique mechanisms to take over the metabolic processes of the host or shut off essential host functions (Koerner and Snustad, 1979; Samson et al., 2013). Current research efforts are mainly focused on the following four types of phage derived host shut-off proteins: (1) The phage enzymes dissolving the bacteria directly, such as peptidoglycan hydrolases, endolysin, and holin, are encoded by late genes (Hermoso et al., 2007); (2) Some early and/or delayed early proteins interacting with global regulators of bacteria, for example AsiA, MotA, and Mrh of T4 phage, which interact with σ^70^ or σ^32^ of bacterial RNA polymerase (Orsini et al., 1993; Ouhammouch et al., 1995; Mosig et al., 1998); (3) Proteins inhibiting bacterial DNA replication, such as the protein O of λ phage and gp79 of phiEco32 phage (Liberek et al., 1988; Savalia et al., 2008). (4) Proteins disturbing the metabolism of bacteria, for instance, gp1.2 of T7 phage inhibiting dGTPase of *Escherichia coli* by combination (Huber et al., 1988). In addition to these, a current study reported that the gp0.4 of T7 phage inhibited bacterial cell division by binding FtsZ directly (Kiro et al., 2013). Extensive application of antibiotics accelerates the emergence of resistance to existing antibiotics and creates multidrug-resistant bacteria that are difficult to treat (Boucher et al., 2009). Traditional antibiotic discovery efforts have failed to keep up with the evolution of this resistance (Lewis, 2013). These phage-derived inhibitors provide a strategy for the discovery of novel antibacterial agents.

The number of phage is 10 times the number of bacteria in the biosphere, which provides a huge resource pool for exploring potential antibacterial compounds (Brüssow and Hendrix, 2002). However, only approximately 1,400 phages are sequenced so far and most ORFs coded by these phages are functionally unknown genes. Our understanding of phage gene products is only the tip of the iceberg. The knowledge of phage gene products and their targets in bacteria is quite significant in antibacterial agent discovery. For instance, Liu et al identified 31 novel polypeptide families inhibiting the growth of *Staphylococcus aureus* from 26 *S. aureus* phage and 11 small molecule compounds mimicking the growth-inhibitory effect of phage products were further screened as potential antimicrobial agents (Liu et al., 2004). In our current work, genome-wide interactions between phage PaP3 and *Pseudomonas aeruginosa* PA3 showed that 38% (2160) host genes were differentially expressed, 98% (2120/2160) of which were down-regulated genes. The co-expression network between phage PaP3 and the host suggested that the early genes of PaP3 had the primary responsibility for the expression inhibition of host genes (Zhao et al., 2016). However, all of the early genes of PaP3 have not been assigned specific functions.

In this study, one early gene product of PaP3 was shown with growth-inhibitory effects on *P. aeruginosa* and *E. coli*, which was encoded by *orf70.1* and called gp (**g**ene **p**roduct) 70.1. Further investigation of the effects of gp70.1 on *P. aeruginosa* PA3 by gene array, RT-qPCR, nuclear magnetic resonance (NMR) spectroscopy and phenotype analysis suggested that the bacterial metabolism and extracellular functions were mainly affected by gp70.1. Finally, bacterial two-hybrid (B2H) and GST pull-down assays suggested the RpoS was a cellular target of gp70.1. In *P. aeruginosa*, RpoS is a global stress response regulator that controls the expression of genes which confer resistance to various stresses, such as starvation, heat shock, osmolarity, antibiotic, and oxidative stress. In addition, it acts as a global factor that controls quorum-sensing gene expression and a regulator of the exoproducts implicated in the virulence of *P. aeruginosa* (Suh et al., 1999; Schuster et al., 2004). Interactions between gp70.1 and RpoS caused the shutoff of the bacterial functions regulated by RpoS, which could be the basis for the subsequent study of antimicrobial agents and targets.

## Materials and Methods

### Bacterial Strains, Plasmids, and Primers

The bacterial strains and plasmids used in this study are listed in **Table 1**, and the primers are listed in **Table 2**. *Escherichia coli* and *P. aeruginosa* strains were grown in Luria-Bertani (LB) medium (for broth culture) or 1.5% (wt/vol) agar LB plates at 37°C. Antibiotics were used at the following concentrations (μg/mL): ampicillin (Amp; 100) and gentamicin (Gm; 20) for *E. coli* and *P. aeruginosa*.

**Table 1:** Plasmids and strains.

Strain	Relevant characteristics or purpose	Reference
***E. coli* strain**		
DH5α	Gene clone	Laboratory collection
BL21(DE3)	Protein expression	Novagen
KS1	Bacterial-two hybrid reporter	Laboratory collection
***P. aeruginosa* strains**		
PA3	A clinical isolate strain; wild-type	Laboratory collection
PA3/ctrl	PA3 bearing plasmid pUCP24	This study
PA3/*orf70.1*	PA3 bearing plasmid pUCP24-*orf70.1*	This study
**Plasmids**		
pUCP24	Plasmid for expression construct; GmR	Laboratory collection
pUCP24-*orf70.1*	pUCP24 with the *orf70.1* gene; GmR	This study
pET-22b	Plasmid for expression construct; GmR	Laboratory collection
pET-22b-*orf70.1*	pET-22b with the *orf70.1* gene with C-terminally His-tagged; AmpR	This study
pGEX-6p-1	Plasmid for expression construct; AmpR	Laboratory collection
pGEX-6p-1-flgM	pGEX-6p-1 with the *flgM* gene with N-terminally GST-tagged; AmpR	This study
pGEX-6p-1-*rpoS*	pGEX-6p-1 with the *rpoS* gene with N-terminally GST-tagged; AmpR	This study
pRBR	Bacterial two-hybrid assay; AmpR	Rao et al., 2009
pRBR-*orf70.1*	pRBR with the *orf70.1* gene; AmpR	This study
pRAC	DNA library construction for bacterial two-hybrid assay; CmR	Rao et al., 2009
pACλCI	P*_lacUV 5_*-directed synthesis of the λCI protein	Dove et al., 1997
pACλCI-β-flap	Positive control for bacterial two-hybrid assay	Rao et al., 2009
pBRL28	Positive control for bacterial two-hybrid assay	Rao et al., 2009

**Table 2:** Primers used in this work.

Gene	Forward Primer (5′–3′)	Reverse Primer (5′–3′)	Purpose
*16S rRNA*	CAAAACTACTGAGCTAGAGTACG	GCCACTGGTGTTCCTTCCTA	Real-time PCR
*orf70.1*	ACCAGGGTTTCTCTGCGG	GGTTCGTAGACAACGGCAAG	
*pilI*	GGTGGAGCACCTGGACGT	TGGAAGACGCCATGAATGA	
*fliC*	GACCAACATCTCGGAAAACG	ACCTGGTTCTTCGACAGCG	
*ffh*	GATCCGTTCCATCCCGAC	AGCCCTTGCCCTTCTTGA	
*cypS*	CTATCTCTCCGCCTGGGG	TGAACAGCTTGCCGACGA	
*arcC*	AAAGCCACCACTTCCTTCTCT	CGATTTCGTTGAGATACAGCTG	
*sucA*	TGTGGAACAGTGCCCATCTAT	TACGTGCGCCACTCTTCTG	
*rplR*	GCCTCGACCCTGGACAAA	GGCGACCAGTTGACCAACT	
*orf70.1*	CGCGGATCCTTGATCGAGGGAGAACTC	CGCCATATGTCACTGTCGAAGTAAACAAC	Clone construction
*orf70.1*	CGGGGATCCCTTGATCGAGGGAGAACTCGTC	CCGCTCGAGTCACTGTCGAAGTAAACAACTGA	Bacterial-two hybrid assay
*orf70.1*	CGCCATATGTTGATCGAGGGAGAACTC	CCGCTCGAGTCACTGTCGAAGTAAACAAC	Protein expression
*rpoS*	CCGGAATTCATGGCACTCAAAAAAGAAGG	GCGTCGACTCACTGGAACAGCGCGTCACTC	
*flgM*	CGCGGATCCATGGTCATCGACTTCAACCGGCT	TCCCCCGGGTCAGCGCTGGGATTCGAAGT	

### Microarray Analysis and RT-qPCR

For both microarray analysis and RT-qPCR, total RNA was isolated from lag phase (2 h), logarithmic phase (7 h) or stationary phase (15 h) cultures of PA3/ctrl and PA3/*orf70.1* by SV Total RNA Isolation System (Promega, USA). In RT-qPCR analysis, the synthesis of cDNA was prepared with a PrimeScript RT reagent kit (TaKaRa Bio; Dalian, China) according to the manufacturer’s recommendations. Quantitative real-time PCR was performed using SYBR Premix Ex Taq II (TaKaRa Bio). Primers used in this study are listed in **Table 2**. *16S rRNA* was selected as the reference gene for normalization. The raw data of the microarray experiments was deposited in the GEO database^1^ with accession number GSE77297.

### Physiological and Biochemical Tests of Bacteria

A transmission electron microscope was employed for the bacterial morphology with 2% *p* – tungstic acid. Laser Scanning Microscope (Zeiss, model LSM 510; Jena, Thuringia, Germany) was used to observe the proliferation of bacteria. The twitching/swimming-motility assay was performed by stabbing a colony of bacteria into the bottom of a petri plate containing 10 ml of agar medium (1% agar content for twitching and 0.3% for swimming). Following incubation at 37°C for 17 h, the zone of motility was measured (Alm and Mattick, 1995). Sensitivity to hydrogen peroxide (H_2_O_2_) was measured as previously described (Hassett et al., 1995). For sensitivity to PaP3, bacterial cultures in mid-log (OD600 = 0.3) were placed on LB plates, and 1 μl PaP3 (10^9^ pfu/ml) was dropped on the bacteria-seeded plates and incubated at 37°C for 18h, and the zones of plaque were measured. For antibiotic sensitivity tests, *P. aeruginosa* PA3/ctrl or PA3/*orf70.1* were tested against 11 antimicrobial agents: piperacillin, netilmicin, ceftazidime, aztreonam, meropenem, polymyxin, amikacin, ciprofloxacin, tobramycin, cefepime, and imipenem. Antimicrobial sensitivity was monitored by Kirby-Bauer disk diffusion assay with commercially available disks (Bio-Rad) (Bauer et al., 1966).

### Bacterial Two-Hybrid Assay

To identify proteins from *P. aeruginosa* that could interact with gp70.1, we used the bacterial two-hybrid system (Dove et al., 1997). Genomic DNA was isolated from *P. aeruginosa* strain PA3 and partially digested with *Sau*3AI. Sau3AI fragments (∼ 500–1,500 bp) were gel purified and ligated into *Bam*HI-digested pRAC. This plasmid library was transformed into *E. coli* strain KS1 containing plasmid pRBR-*orf70.1* or pRBR (negative control). As a positive control, we used plasmids pBRL28 and *p*ACλCI-β-flap (Rao et al., 2009). Co-transformants were then placed on LB plates supplemented with 100 μg/mL of Amp, 50 μg/mL of kanamycin, 34 μg/mL of chloromycetin (Cm), 0.1 mM of IPTG, and 50 μg/mL of X-gal. Plates were incubated at 30°C for 24 h, and then β-galactosidase assays were performed as previously described (Thibodeau et al., 2004).

### GST Pull-Down Assay

His-tagged gp70.1, GST-tagged FlgM, GST-tagged RpoS and GST alone were purified with Ni-agarose or glutathione-agarose. GST pull-down assays were performed by Pierce GST Protein Interaction Pull-Down kit (Thermo Scientific, USA) according to the manufacturer’s instructions. Samples were separated by SDS-PAGE, stained with Coomassie blue, and the captured His-gp70.1 was detected by a monoclonal antibody against the His tag.

### Sample Preparation and NMR Spectroscopy

Bacteria were cultured for 7 h and 2 mL media was placed in the ice for 20 min, followed by centrifugation at 15, 000 × *g* for 10 min at 4°C. After filtering the bacteria onto the membrane (0.22 μm), 1 mL of supernatant per sample was stored at -80°C until NMR analysis. For NMR analysis, 450 μl supernatant per sample was added with 50 μl Anachro Certified DSS Standard Solution (ACDSS, 4.088 mM) and centrifuged at 13, 000 × *g* at 4°C for 2 min before transferred into the NMR tubes. All the NMR experiments were performed on an Agilent DD2 600 MHz spectrometer (Agilent Technologies, Santa Clara, CA, USA) equipped with a triple-resonance cryogenic probe at 298.15 K. Metabolites were identified and quantified using Chenomx NMR Suit (version 8.0, Chenomx, Edmonton, AB, Canada). Prior to Fourier transformation, phasing and baseline correction, a line broadening of 0.5 Hz was applied to the free induction decay. The full width at half maximum (FWHM) of the DSS-d6 peak at 0.00 ppm was assessed for each spectrum. A total of 49 metabolites were obtained from the Chenomx database containing more than 250 compounds. The concentrations of the metabolites in each spectrum were normalized by the absolute spectral intensity.

### Animal Experiment

The animal experiment was approved by the Animal Research Ethics Committee of Third Military Medical University, China. *P. aeruginosa* were grown in LB medium at 37°C until the early stationary phase and were collected by centrifugation at 10,000 × *g* for 1 min. The cell pellet was washed twice with saline buffer and resuspended in the same buffer at a final concentration of 3 × 10^7^ cfu/ml. Each of two groups of 10 mice (6–8-week-old BALB/c female mice) was injected intraperitoneally with 1ml of PA3/*orf70.1* or PA3/ctrl, followed by 24 h of observation. Three independent experiments were performed.

### Data Analysis and Visualization

For microarray dataset, PseudoCAP (Pseudomonas aeruginosa Genome Database) function analysis was used to assess the functions of the DEGs (differentially expressed genes) (Winsor et al., 2005). Package “OmicCircos” in R was used for the generation of circular plots (Hu et al., 2014). The software STEM (Short Time-series Expression Miner) was applied for the comparison and visualization of the short time series (2, 7, and 15 h) gene expression datasets between PA3/*orf70.1* and PA3/ctrl (Ernst and Bar-Joseph, 2006). STEM analysis uses a correlation-based clustering algorithm. Briefly, 49 distinct and representative model profiles of temporal expression were selected independent of the data. Each gene of microarray dataset was assigned to the model profile that most closely matched the gene’s expression profile by the correlation coefficient (*r*). The algorithm then determined which profiles have a statistically significant higher number of genes assigned using a permutation test (*p*).

In NMR metabolomics, software MestReNova 9.0 (MestreLab, Santiago de Compostela, Spain) was used to obtain peak areas from the raw spectrum. Principal Components Analysis (PCA) and Partial Least Squares Discriminant Analysis (PLS-DA) were performed by PCA Methods Bioconductor and pls package in R. For each metabolite, the Variable Importance in the Projection (VIP) score was calculated to determine the most relevant metabolites in phage infection (Chong and Jun, 2005). The R’s ggplot2 was used for data visualization.

## Results

### Growth Inhibitory Effects of Bacteriophage PaP3 Gp70.1 on Bacteria

**FIGURE 1:**
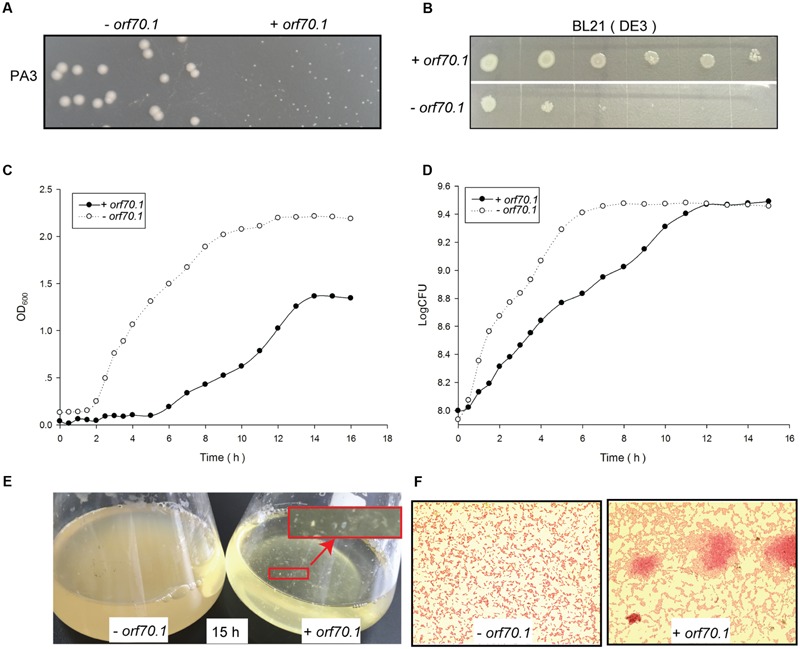
**Inhibition effects of gp70.1 on bacterial growth. (A)** PA3 colonies on LB agar plate. **(B)** Inhibition of gp70.1 to *E. coli* BL21 (DE3). *E. coli* was 10-fold diluted serially and then inoculated on LB agar supplemented with 1mM IPTG. **(C)** Growth curve of PA3 based on absorbance value of OD_600_. **(D)** Growth curve of PA3 based on colony forming units. **(E)** Bacterial culture in LB broth cultured for 15 h. The red sign showed macroscopic cell aggregates of PA3/*orf70.1*, which were also observed in Gram stain. **(F)** Gram stain of bacteria cultured for 15 h. “- *orf70.1*” shows bacteria transformed with an empty plasmid, while “+ *orf70.1*” shows bacteria with plasmid carrying *orf70.1*.

Aimed to screen inhibitors from early gene products of PaP3, early genes were cloned into expression vector pUCP24. *P. aeruginosa* PA3 were transformed with early gene expression vectors (PA3/*orf*) or the empty vector (PA3/ctrl) and the changes of colony phenotype were first observed. Then, a dwarf colony was observed in PA3 containing *orf70.1* (PA3/*orf70.1*), which showed a needle-like colony and a little secretion around the colony compared to the control (**Figure 1A**). To evaluate the influence of the gene products (gp70.1) of *orf70.1* on PA3, the growth efficiency of bacteria was further examined by growth curves based on optical cell densities at 600 nm (OD600) measurement (**Figure 1C**) and CFU (colony-forming unit) calculation (**Figure 1D**). Gp70.1 had a severe inhibitory effect on the concentration of bacterial culture. Intriguingly, the growth curve based on CFU showed that the growth of PA3 was delayed by gp70.1 and the number of living bacteria was similar with the control in the stationary phase. At the same time, macroscopic cell aggregates of PA3/*orf70.1* appeared in the culture, which were also observed in Gram staining (**Figures 1E,F**). This might be a reason for the low OD values caused in the absence of gp70.1. Moreover, the growth-inhibitory effect of gp70.1 was non-specific so it also worked in *E. coli* (**Figure 1B**).

### Features of *orf70.1* and Gp70.1

**FIGURE 2:**
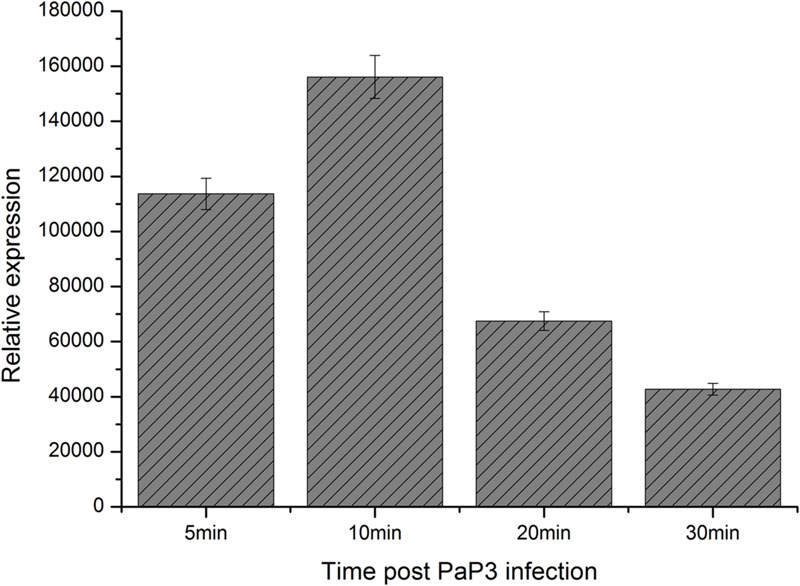
**RT-qPCR analysis for detecting the relative expression of *orf70.1* during infection of host bacteria by PaP3**.

The gene *orf70.1* was first recognized as a probable gene by GeneMark and showed a high expression level at the early stage of phage infection in the RNA-seq analysis (data to be published separately). This gene was located on the positive strand, beginning at position 44,741 and ending at position 45,133 (393 bp). According to the one-step growth curve of phage PaP3, RT-qPCR analysis was used to examine the expression of *orf70.1* at different infection periods. The results also showed a high expression level of *orf70.1* at 5 min and reached a peak value at 10 min (**Figure 2**), which confirmed that the *orf70.1* was an early gene.

The gene product of *orf70.1* (gp70.1) was predicted to consist of 131 amino acids. A similarity search was run by BlastP against non-redundant protein sequences and no putative conserved domains were detected in gp70.1. In the BlastP analysis, 8 similar sequences (hits) were found. The top two hits were metallophosphoesterase (*e*-value = 0.57, ident = 38%) and anti-anti-sigma factor (*e*-value = 1.4, ident = 38%). The purified gp70.1 was obtained from BL21 (DE3)/pET22b cells. The molecular weight of gp70.1 was approximately 13 kd and consistent with the prediction according to SDS-PAGE. The record of the Pseudomonas phage PaP3 RefSeq Genome (GenBank: AY078382.2) was updated based on this study.

### Microarray Analysis of the Effects of Gp70.1 on *P. aeruginosa*

**FIGURE 3:**
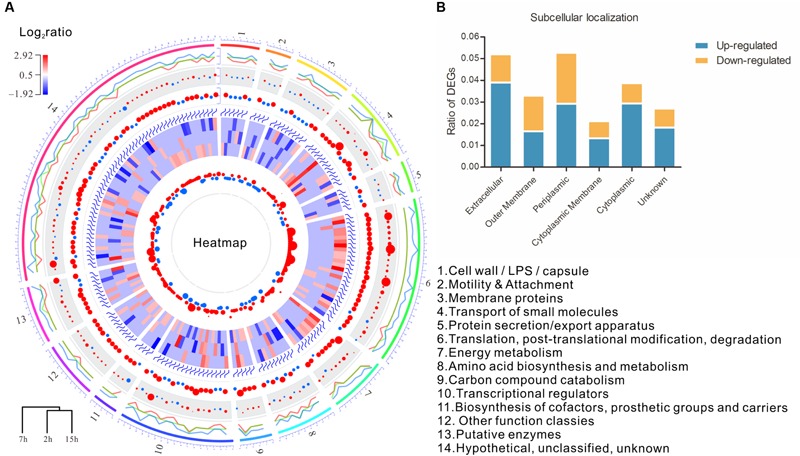
**Microarray analysis reveals influence of gp70.1 on gene expression of PA3. (A)** Circular plots generated by OmicCircos showing the expression and function of DEGs. A total of 178 DEGs and 14 terms (1–14) of PseudoCAP function were involved. Circular tracks from outside to inside: track 1 shows the 14 PseudoCAP functions showed with different colors numbered with 1 to 14; track 2 is the three lines for quantile values for the detected fold changes (log_2_ratio) of gene expression ranged from -1.9 to 2.9. The middle line is for the median, the outside line and the inside line are for 90 and the 10%, respectively; Track 3 is the circle points with the center = median and radium = variance; Track 4 is the circle plot with the center equal to the mean and scaled value; Tracks 5 is the heatmap of 178 DEGs (red: up-regulated, blue: down-regulated); Track 6 is the circle plot with the center = median and radius = standard deviation; Track 7 is the 95% confidence interval of the fold changes. **(B)** Subcellular localization of DEGs. The percentages of up- or down-regulated genes in each subcellular localization were calculated and shown on *y*-axis.

The early genes of phage usually encoded regulatory proteins to control the expression of host genes. Based on the growth curve of PA3/*orf70.1* and PA3/ctrl, three time points containing lag (2 h), logarithm (7 h), and stationary phase (15 h) were chosen to compare the whole gene expression profile of PA3 with or without gp70.1 at each time point. Compared to the control (PA3/ctrl), a total of 178 genes of PA3 were differentially expressed (ratio > 2, *p* < 0.05) in the presence of gp70.1, of which, 40 DEGs(differentially expressed genes) were detected at 2 h, 79 DEGs at 7 h and 75 DEGs at 15 h. As shown in **Figure 3A**, 14 PseudoCAP (Pseudomonas aeruginosa Genome Database) function terms were attributed to the 178 DEGs, which could be divided into two main categories: extracellular functions and metabolism functions (Winsor et al., 2005). About one third of the DEGs were involved in the extracellular function including cell wall/LPS/capsule, motility and attachment, membrane protein, transport of small molecules and protein secretion/export apparatus. In addition, subcellular localization of the DEGs in **Figure 3B** suggested that “periplasmic” and “extracellular” locations contributed larger proportions of DEGs. The metabolism functions were related to energy, amino acid and carbon compound metabolism. Moreover, a large number of genes related to translation were up-regulated at 7 h and 54% of them were genes encoding ribosomal proteins.

**FIGURE 4:**
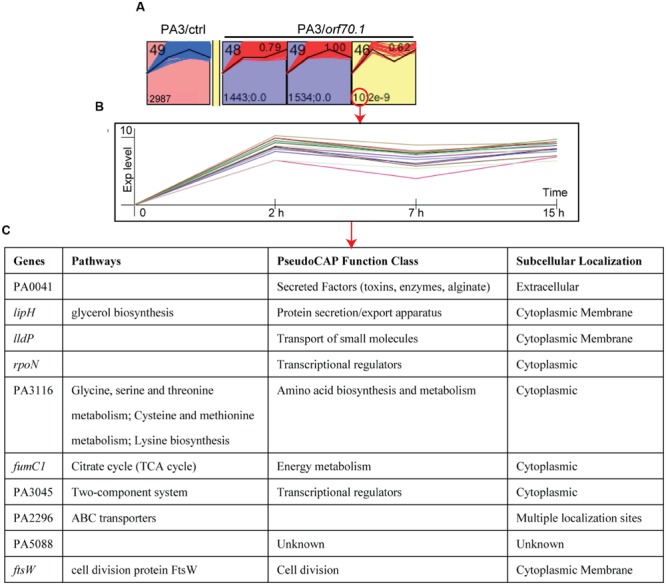
**STEM (Short Time-series Expression Miner) analysis of microarray data.** A total of 49 model profiles are ordered by ID (top left). Each gene of PA3 was assigned to different model profiles based on the time series expression values (log ratios) from different experimental conditions (PA3/ctrl or PA3/*orf70.1*). **(A)** Profiles with significant difference between the original set profile (PA3/ctrl) and comparison set profile (PA3/*orf70.1*). The first expression values were transformed into 0. The expression pattern containing 2,987 PA3 genes was changed from profile 49 into profile 48 (1,443 genes, *p* = 0), 49 (1,534 genes, *p* = 0) and 46 (10 genes, *p* = 2*e* - 9) when overexpressed gp70.1 in PA3. Profile 46 shows the minimum correlation (*r* = 0.62, upper right). **(B)** Detailed model profile information of the most changed 10 genes in profile 46. **(C)** The information of pathway, function, and subcellular localization of the 10 genes.

Since the growth of PA3 were delayed in the presence of gp70.1, the comparison at each time point did not eliminate the influence caused by the different growth state between PA3/ctrl and PA3/*orf70.1.* In order to stringently examine the effects of gp70.1 on the gene expression of PA3, the Short Time-series Expression Miner (STEM) was used to complementarily analyze the microarray data (Ernst et al., 2005). In STEM analysis, 49 temporal expression profiles were selected as model profiles, which were ordered from 1 to 49 (**Figure 4A**, top left). Each gene of PA3 was assigned to different model profiles based on the time series expression values (log ratios) from different experimental conditions (PA3/ctrl or PA3/*orf70.1*). A total of 2,987 genes from PA3/ctrl were assigned to profile 49, while these genes exhibited three patterns of expression: profile 49 (1,534 genes, *p* = 0), 48 (1,443 genes, *p* = 0) and 46 (10 genes, *p* = 2*e* - 9) when gp70.1 was overexpressed in PA3. The minimum of the correlation (*r*) of 2 profiles was 0.7 in STEM analysis, so that the expression patterns of 10 genes in profile 46 (*r* = 0.62) were significantly changed by gp70.1. Compared to the model profile 49, the significant difference of these genes in profile 46 was mainly reflected in the reduction at 7 h (**Figure 4B**). PseudoCAP functions and subcellular localizations of the 10 genes in **Figure 4C** showed that three genes (PA0041, *lipH*, and *lldP*) were related to the extracellular function and four genes (PA0041, *lipH, lldP*, and *ftsW*) were located in extracellular locations (Winsor et al., 2005). Notably, RNA polymerase sigma factor RpoN that positively regulating bacterial nitrogen metabolism and motility also appeared to have a changed expression pattern in the presence of gp70.1 (Totten et al., 1990). Thus it can be seen, *orf70.1* might encode a regulatory product, which caused differentially expressed genes mainly related to extracellular function and metabolism of *P. aeruginosa*.

### RT-qPCR Validation of Selected DEGs

**FIGURE 5:**
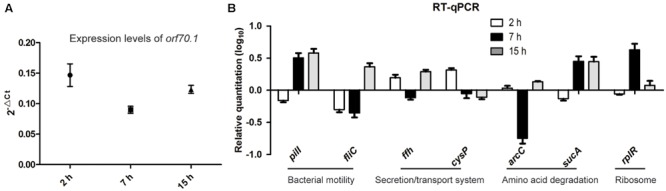
**RT-qPCR validation of selected DEGs from microarray data set. (A)** Relative expression level of *orf70.1* in PA3 was shown with 2^-Δ^ ^Ct^ (ΔCt = Ct *orf70.1* – Ct *16S rRNA)*. **(B)** RT-qPCR analysis of the selected seven DEGs at the different time point. The qPCR results were normalized using *16S rRNA* and expressed as fold change (Log_10_ scale) by the comparative Ct method. Control (PA3/pUCP24-2 h, 7 h, and 15 h) is normalized as 0.

Although gp70.1 caused inhibited growth of PA3, there were unexpectedly more activated genes than inhibited genes at each time point in our microarray analysis. Seven DEGs selected from microarray data were validated by real-time qPCR (**Figure 5**), as being related to bacterial motility (*pilI* and *fliC*), secretion and transport (*ffh* and *cypS*), anima acid metabolism (*arcC* and *sucA*) and ribosome protein (*rplR*). The expression levels of *orf70.1* in *P. aeruginosa* were also examined. The results showed that *orf70.1* was stably expressed at each growth stage with the *C*t values ranging from 16 to 17 (**Figure 5A**). The RT-qPCR results showed a consistent directional change compared to microarray assay. All the 7 detected DEGs were shown with varying degrees of upregulation at different time points. The expression of the twitching motility protein PilI gene (*pilI*) and flagellin type B gene (*fliC*) were reduced at 2 h and both up-regulated at 15 h (**Figure 5B**). Meanwhile, *ffh* and *cysP*, related to protein export and sulfate transport were activated at the lag phase. Remarkably, the RT-qPCR results confirmed the differential expression of the genes involved in amino acid metabolism, such as *arcC* (carbamate kinase) and *sucA* (2-oxoglutarate dehydrogenase), which were related to arginine deiminase pathway and lysine degradation, respectively. The serious inhibition of *arcC* at the logarithmic phase might hinder arginine utilization of bacteria, while the over-expression of *sucA* would increase lysine consumption. In addition, the significant upregulation of *rplR* at 7 h was consistent with the microarray results.

### The Effects of Gp70.1 on the Metabolome of *P. aeruginosa* by Nuclear Magnetic Resonance (NMR) Analysis

**FIGURE 6:**
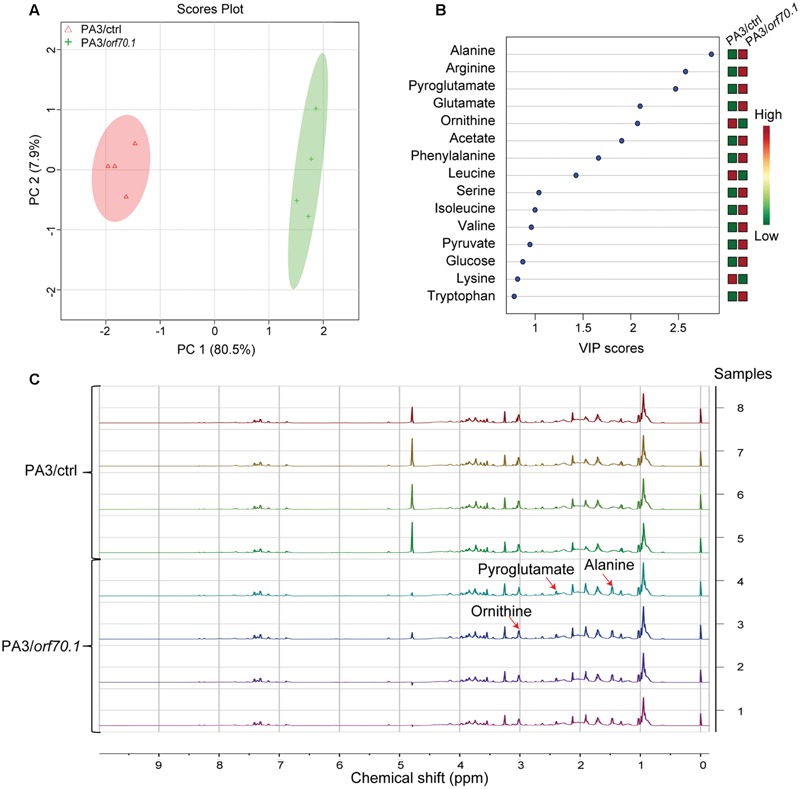
**Influence of gp70.1 on the metabolite consumption of PA3. (A)** Score plot of PCA (Principle Component Analysis) of the normalized NMR data from eight samples. It was constructed based on the first two principle components (PC1 and PC2). PC1 corresponds to 80.5% of total variance and PC2 corresponds to 7.9%. **(B)** Variable importance in projection (VIP) analysis is used to show the significantly changed metabolites. The higher VIP-score means the more significant difference. The concentrations of metabolites are color-coded: red for high and green for low concentration. **(C)** ^1^H-NMR spectra of eight samples (four biological replicates in each group). NMR spectra were reported with chemical shift range from 0 to 10 ppm. Three peaks assigned to three differential metabolites (alanine, pyroglutamate, and ornithine) were illustrated by red arrows.

Based on the initial observation that the OD values of the bacterial culture were reduced and the differentially expressed genes were related to bacterial metabolism in the presence of gp70.1, the metabolites in bacterial supernatant at the logarithmic phase were detected by NMR spectra. There were a total of eight samples – four biological replicates for PA3 with overexpressed gp70.1 (PA3/ctrl) and 4 biological replicates for the control group (PA3/*orf70.1*). The eight samples in principle component analysis (PCA) diagram were distinctly separated into two groups, indicating the difference of supernatant components between PA3/ctrl and PA3/*orf70.1* (**Figure 6A**). Finally, 15 significantly differential metabolites, including 12 amino acids, two organic acids, and one sugar, were identified by Variable Importance in Projection (VIP) analysis (**Figure 6B**). The contribution of metabolites in distinguishing the sample classification was measured by VIP score. The top five metabolites (alanine, arginine, pyroglutamate, glutamate, and ornithine) with VIP scores of more than two all belonged to amino acids. Most (9/12) of the differential amino acids were shown with higher concentrations in PA3/*orf70.1* than the control. It meant that the main effects of gp70.1 on the metabolism of PA3 were the reduction of amino acid consumption. **Figure 6C** presents three significantly differential metabolites: alanine and pyroglutamate with the higher concentrations and ornithine with a lower concentration. Furthermore, the NMR results confirmed RT-qPCR analysis: the down-regulated *arcC* did reduce arginine uptake, while the activation of *sucA* increased lysine consumption.

### Phenotype Analysis of the Effects of Gp70.1 on *P. aeruginosa*

**FIGURE 7:**
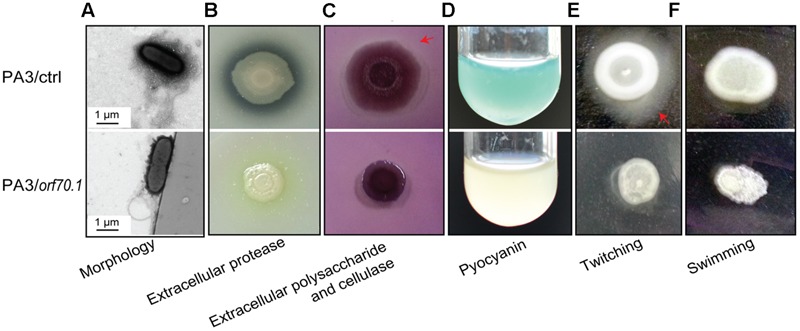
**Phenotypic analyses of *P. aeruginosa* (PA3/ctrl and PA3/*orf70.1*). (A)** Transmission electron microscope image. **(B,C)** Detection of extracellular protease, polysaccharide and cellulase of *P. aeruginosa* on LB plates containing 2% milk or 0.1 congo red. **(D)** Pyocyanin of *P. aeruginosa* in LB medium. **(E,F)** Motility experiments on LB plates with 1% agar for twitching and 0.3% agar for swimming.

To further demonstrate the effects of gp70.1, a series of phenotype experiments were performed on the basis of the above results. First, the transmission electron microscope image showed no difference of morphology between PA3/ctrl and PA3/*orf70.1* (**Figure 7A**). However, it was obvious that PA3/*orf70.1* showed fewer secretions around the cell compared to PA3/ctrl. In addition, we further found that gp70.1 severely inhibited the measured extracellular metabolites of *P. aeruginosa* including protease, polysaccharide, extracellular cellulase, and pyocyanin (**Figures 7B–D**). The genes (*pilI* and *fliC*) related to motility were down-regulated in the microarray assay and RT-qPCR analysis. Thus, bacterial motility was examined on LB agar plates (1% agar for twitching and 0.3% agar for swimming). The results showed a strong inhibitory effect of gp70.1 on the motility of *P. aeruginosa* (**Figures 7E,F**). At this point, it was clear that gp70.1 exerted broad inhibitory effects on extracellular functions and metabolism, which caused multiple repressed phenotypes of *P. aeruginosa.*

### Identification of the Cellular Targets of Gp70.1

**FIGURE 8:**
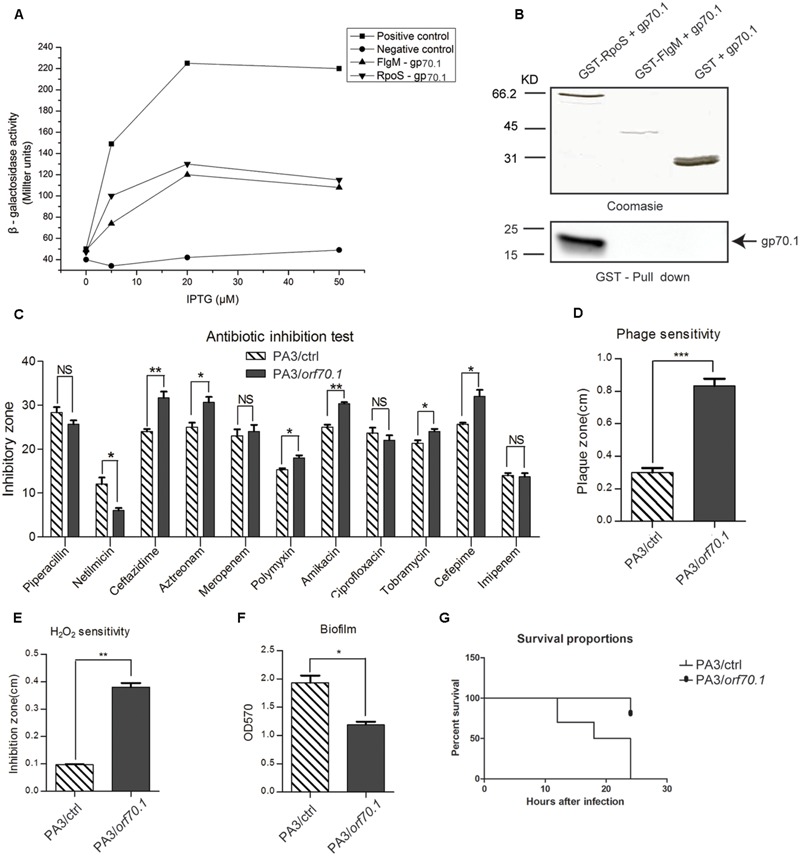
**Identification of the target protein of gp70.1. (A)** Bacterial two-hybrid assay was used to screen target proteins of gp70.1 in PA3, showing FlgM and RpoS the potential targets of gp70.1. **(B)** Confirmation of the FlgM-gp70.1 and RpoS-gp70.1 interactions by GST Pull-down. **(C–G)** Evaluation for the influence of gp70.1 on the typical biological function regulated by RpoS. The statistical significance of changes in expression was assessed by paired *t*-test (^∗^*p* < 0.05; ^∗∗^*p* < 0.001; ^∗∗∗^*p* < 0.0001).

We hypothesized that gp70.1 exerted the inhibitory effects on *P. aeruginosa* by the interactions with its cellular targets. To track this, a bacterial two-hybrid (B2H) assay was used to screen the targets of gp70.1 in the PA3 genomic library. Two potential target proteins, FlgM and RpoS, of gp70.1 were finally identified (**Figure 8A**). To validate the B2H results, pull-down experiments were performed using His-tagged gp70.1, GST-tagged FlgM and GST-tagged RpoS. The results indicated that the RpoS could interact with gp70.1 directly *in vitro*, while no interaction was detected between gp70.1 and FlgM (**Figure 8B**). As a global regulator, RpoS is involved in the regulation of broad functions in *P. aeruginosa* including: biofilm, motility, virulence, antibiotic resistance and, most primarily, the stress response. We then tested these RpoS-regulated functions of PA3/ctrl and PA3/*orf70.1*. Antibiotic sensitivity tests were performed with 11 antibiotics (**Figure 8C**), the results indicated that gp70.1 increased the sensitivity of PA3 to 6 antibiotics: ceftazidime, aztreonam, polymyxin B, amikacin, tobramycin, and cefepime, while it decreased the sensitivity to netilmicin. In the phage and H_2_O_2_ sensitivity experiments, PA3 containing gp70.1 showed a higher sensitivity than the control group (**Figures 8D,E**). Furthermore, biofilm formation of PA3 was significantly inhibited by gp70.1 (**Figure 8F**). Lastly, and most importantly, gp70.1 greatly reduced the virulence of PA3 so that the survival rate of mice vaccinated with PA3/*orf70.1* was up to 80% within 24 h of observations, but 0% in the case of PA3/ctrl (**Figure 8G**). These results suggested that the interactions of gp70.1 and RpoS had an inhibitory effect on the functions regulated by RpoS.

## Discussion

As we all know, phage is a virus which infects bacteria and the reproduction of phage in the bacterial cell is often lethal. Therefore, the phage-encoded growth inhibitors of bacteria provided us with a clue for an antibacterial strategy. In this work, one such phage protein – gp70.1 encoded by *orf70.1* of *P. aeruginosa* phage PaP3 was identified with a non-specific inhibitory effect on the growth of both PA3 and BL21 (DE3). Moreover, it also worked in PAO1 and DH5α (data not shown). However, the colony phenotype of *E. coli* was not obviously changed in the presence of gp70.1. This was not rare in other phage proteins, such as gp77 and gp78 encoded by mycobacteriophage L5, which were toxic to both *Mycobacterium smegmatis* and *E. coli* (Rybniker et al., 2008). Another study reported that the e3 of phage SPO1 also led to growth inhibition when expressed in *E. coli* (Wei and Stewart, 1993). The strict host specificity of phage is the biggest handicap to the development of phage therapy. The non-specific “shut-off” function observed in gp70.1 indicated that wide-spectrum inhibitors encoded by phage might be a breakthrough to solve this obstacle to phage therapy.

The proliferation of phage in the host cell is needed to minimize the life activity of the host by shutting off most of the metabolism machinery of the cell. As early as 1979, researchers had reviewed the shutoff of host macromolecular (DNA, RNA and protein) synthesis by the phage infection. A survey revealed that most (64%) of the phage toxic proteins were encoded by phage early genes (Roucourt and Lavigne, 2009). The RT-qPCR analysis confirmed the high expression level of *orf70.1* in *P. aeruginosa* during the early infection of PaP3. This meant that gp70.1 might be related to the early events of PaP3 infection, such as host transcription.

In our microarray assay, the up-regulated genes were more than the down-regulated genes when comparing the PA3-expressed gp70.1 with the wide type PA3 at each time point. This was unexpected and illogical to the observed growth inhibition of bacteria. A recent study reported the similar finding that the observed changes in host physiology upon infection failed to correspond with the result of a differential expression of host genes induced by the phage (Chevallereau et al., 2016). Hereby, perhaps one of the reasons for the contradiction could be that the negative effects of gp70.1 on host growth and metabolism were not primarily mediated through down-regulated gene expression but disturbed gene expression. We inferred gp70.1 was used by PaP1 to redirect cellular transcription toward viral reproduction through both activation and inhibition of host gene expression. Another reason could be the result of the delayed growth caused by gp70.1 according to the growth curve of PA3. At the time point of 7 h, PA3/*orf70.1* was at the logarithmic phase of rapid cell growth, while PA3/ctrl was at the stable phase of limited or no growth. A large number of proteins were synthesized for the rapid multiplication of bacteria in the logarithmic phase. This could be an explanation for the massive DEGs involved in the function of translation in microarray analysis. A STEM analysis based on a time-series method was further performed to complement the DEGs analysis at each time point. In both cases, gp70.1 was shown to have a main effect on the genes involving extracellular functions. Moreover, an RNA polymerase sigma factor, RpoN, was observed with a changed expression pattern in the presence of gp70.1. RpoN governs the gene expression of nitrogen metabolism, motility, and attachment. A current study revealed RpoN as the central player within the crosstalk consisting of the 10 most frequent sigma factors in *P. aeruginosa* PA14 (Schulz et al., 2015). Therefore, the changed expression pattern of RpoN caused by gp70.1 could have a broad effect on the host cell, which even extends to other sigma factors crosstalking with RpoN.

We furthermore uncovered that the interference of gp70.1 with the host genes related to extracellular functions and mechanism caused the suppression of the corresponding phenotypes. The amino acid uptake of bacteria was flexible and changed with the environment (Zhu et al., 2007). In this study, the absorption of multiple amino acids was decreased when gp70.1 was expressed in *P. aeruginosa*. In addition, the sugar consumption of bacteria was also reduced in the culture. Since the metabolism of the amino acid and sugar provided the main energy sources for bacterial growth, the loss of these energy materials would inevitably lead to nutritional deficiencies, and thus there appeared a dwarf colony in the case of PA3/*orf70.1*.

The motility of *P. aeruginosa* containing gp70.1 was almost lost completely according to our observation of twitching and swimming. Nevertheless, the flagella PA3/*orf70.1* were visible in the transmission electron microscope image and the genes encoding flagella or pilus were not sharply down-regulated. This implied that the deficiency of bacterial motility was irrelevant to the motor structure. Clearly, one reason was the limit of energy materials, because the motility was energy consuming. Another reason could be the changed expression pattern of RpoN, which was an important regulator of the bacterial motility. Beyond these, the extracellular polysaccharides can also mediate the social motility of *P. aeruginosa* (Li et al., 2003). Thus, the inhibition of gp70.1 to the extracellular polysaccharides should be partly responsible for the deficiency of motility.

For phage, the interactions between phage and host play an important role in taking over the cellular machinery. The binding of phage proteins may have an effect of inhibition or activation of the host proteins. In this work, we showed the direct interaction of gp70.1 and RpoS. In *P. aeruginosa*, RpoS was a well known stress response regulator and the RpoS mutant *P. aeruginosa* showed increased sensitivity to osmotic pressure, high temperature and hydrogen peroxide (Suh et al., 1999). Similar results were observed in *P. aeruginosa*-expressed gp70.1 in that gp70.1 repressed the response of *P. aeruginosa* to several examined stress conditions. That meant the binding of gp70.1 exerted an inhibitory effect on RpoS and gp70.1 could be a potential anti-sigma factor. We will confirm this hypothesis in an additional study. Recently, many studies had revealed the interactions between phage proteins and the components of RNA polymerase and cause the inhibition of bacterial transcription (Roucourt and Lavigne, 2009). However, as far as we know, gp70.1 was the first phage protein shown to have interaction with RpoS.

As described above, gp70.1 had extensive inhibitory effects on the phenotype and virulence of *P. aeruginosa* that were mainly regulated by alternative sigma factors. There are more than 26 sigma factors in *P. aeruginosa* including five primary sigma factors (RpoD, RpoS, RpoH, RpoN, and FliA) and more than 21 extracellular cellular function (ECF) sigma factors (Potvin et al., 2008; Llamas et al., 2009). They control the expression of the respective gene set with specific functions. However, these sigma factors interfered with each other (crosstalk), which meant the same biological function could be controlled by two or more sigma factors. In addition, direct crosstalk was mainly involved in genes of chemotaxis, motility/attachment and secreted factors. For this complex regulatory network of sigma factors, the interaction of gp70.1 and RpoS could have an inhibitory effect on numerous functions directly regulated by RpoS or even beyond the control of RpoS. Intriguingly, the observed small colony on solid medium and macroscopic cell aggregates in liquid medium when expressed gp70.1 in *P. aeruginosa* were similar with the rugose small-colony variants (RSCV) isolated from cystic fibrosis (CF) patients (Starkey et al., 2009). Moreover, decreased expression of motility functions was shown in both cases. The RSCV was formed by the regulation of RpoS through Psl-operon (Irie et al., 2010). We thus speculated that there could be a similar mechanism, in which gp70.1 inhibited the growth of *P. aeruginosa*.

The small size of the phage genomes makes the phage produce proteins with multiple functions. One such example was gp59 encoded by phage T4: it acted as the T4 helicase-loading protein, meanwhile preventing the binding of RNA polymerase to DNA by forming a ternary complex with it (Xi et al., 2005; Yuan and McHenry, 2009). We found that the inhibitory effects of gp70.1 on *P. aeruginosa* and *E. coli* were different, and that the effects of gp70.1 on *P. aeruginosa* were extensive. Thus, we hypothesized that gp70.1 might act with multiple functions in the bacterial cell.

This work presented a phage-derived host shut-off protein, which was shown with non-specific inhibitory activity to the growth of *P. aeruginosa* and *E. coli*. The combination of multiple analytical methods allowed a comprehensive evaluation for the impact of gp70.1 on *P. aeruginosa* at both the genetic level and the phenotypic level. Gp70.1 showed strong inhibition to broad-scale functions of *P. aeruginosa*, such as extracellular functions, amino acid metabolism, stress response and virulence. This study preliminarily revealed the biological function of gp70.1 and indicated the potential value of gp70.1 in antibacterial applications. In addition, most early genes of the currently annotated phage are usually conserved in phage and functionally unknown, so that little reference can be provided in blast analysis of the early phage genes with a public database. Therefore, functional dissection of such PaP3 genes has referential value for the study of other phages.

## Author Contributions

The author(s) have made the following declarations about their contributions: FH, YT, and XZ conceived and designed the experiments; XZ, CC, XJ, GH, SLe, SLu, and JW performed the experiments; XZ, WS, LZ, and QN analyzed the data; JW, YZ, and ML contributed reagents/materials/analysis tools; FH, YT, XR, and XZ wrote the paper.

## Conflict of Interest Statement

The authors declare that the research was conducted in the absence of any commercial or financial relationships that could be construed as a potential conflict of interest.

The reviewer YT and handling Editor declared their shared affiliation, and the handling Editor states that the process nevertheless met the standards of a fair and objective review.

## Footnotes

1 http://www.ncbi.nlm.nih.gov/geo
